# Effect of substituting soybean meal with euglena (*Euglena gracilis*) on methane emission and nitrogen efficiency in sheep

**DOI:** 10.1111/asj.13121

**Published:** 2018-10-26

**Authors:** Ashagrie Aemiro, Shota Watanabe, Kengo Suzuki, Masaaki Hanada, Kazutaka Umetsu, Takehiro Nishida

**Affiliations:** ^1^ Department of Life and Food Sciences Obihiro University of Agriculture and Veterinary Medicine Obihiro Japan; ^2^ Euglena Co., Ltd. Tokyo Japan; ^3^ Department of Agro‐environmental Science Obihiro University of Agriculture and Veterinary Medicine Obihiro Japan

**Keywords:** euglena, methane emission, nitrogen efficiency, sheep

## Abstract

This study evaluated methane (CH_4_) emission, intake, digestibility, and nitrogen efficiency in sheep fed diets containing replacement levels (0%, 33%, 50%, and 67% of soybean meal with euglena). In this experiment, four Corriedale wether sheep with an initial body weight of 53.8 ± 4.6 were arranged in a 4 × 4 Latin square design. This experiment lasted 84 days, divided into four experimental periods. Each period lasted 21 days, which consists of 14 days of adaptation to the diets, 5 days to collect samples, and 2 days to collect gas emission from sheep. Methane emission expressed as L/kg DM intake or g/kg DM intake reduced by up to 37% and the energy loss via CH_4_ (% of GE intake) reduced by up to 34%. No differences (*p *>* *0.05) were observed in DM and OM intake and whole tract apparent DM digestibility due to substitution of soybean meal with euglena. The total CP loss reduced significantly (linear, *p *<* *0.001) and CP efficiency increased linearly (*p *=* *0.03) with increasing concentration of euglena. As a result, nitrogen balance and average daily weight gain remained unchanged despite higher nitrogen concentration in soybean supplemented group. In conclusion, substitution of soybean meal with euglena reduced methane emission without affecting the performance of animals.

## 1 INTRODUCTION

Livestock plays an important role in climate changes accounting for an estimated 14.5% of the anthropogenic global greenhouse gas (GHG) emission (Gerber et al., 2013). Methane emission from ruminants (Veysset, Lherm, & Bébin, 2010) or manmade activities (Gerber et al., 2013) is the main GHG that comes during the fermentation of feeds in the rumen. Among the strategies recognized, diet modification appears to be one of the promising methods to reduce methane emission (Rossi et al., 2017). Feed substitutes and supplements are highly effective ways to increase efficiency and change fermentation process in the animal to decrease the intensity of GHG emission. Numerous nutritional and toxicological evaluations have proved the suitability of algal biomass as a feed supplement (Becker, 2004). Microalgae contain several bioactive compounds and various processes have demonstrated the potential of these microorganisms in food, animal feed, cosmetics industries, and the production of pigments and additives (Vaz, Moreira, de Morais, & Vieira Costa, 2016). Microalgae cultivation significantly aids in reducing the greenhouse effect due to its carbon dioxide bio fixation capacity (Brennan & Owende, 2010; Wang, Li, Wu, & Lan, 2008). In addition to this, microalgae do not compete with food crops because they do not require arable land and can use waste as nutrients for development. (Vaz et al., 2016).

Soybean is one of the main oilseeds used for biodiesel production (Koirala, Mishra, D'Antoni, & Mehlhorn, 2015) and its byproduct (soybean meal) is the major protein supplement for livestock. However, the price of this commodity varies from time to time and affects biodiesel price (Koirala et al., 2015), suggesting to look in to an alternative sources to reduce the price of biodiesel. As a result, there is growing interest in using microalgae for biodiesel production, especially because some of the micro algae produce about 150 times more oil per hectare than soybean (Satyanarayana, Mariano, & Vargas, 2011). Microalgae are currently cultivated commercially for human nutritional products and biofuel and the total production is estimated to be 10,000 tons per year (Benemann, 2008). Recent reports indicated that the mass culture and commercial production of microalgae have grown (Sathasivam, Radhakrishnan, Hashem, Elsayed, & Abd_Allah, 2017) and microalgal fuel production cost is relatively close to being economically feasible (Stephens et al., 2010). Production costs can be reduced when using wastewater and flue gases as nutrient sources regardless of the technology but only where regulations allow it (Acién et al., 2017). It has been reported that 30% of the current world algae production is sold for animal feed (Becker, 2004). The use of microalgae in animal feed may potentially offer many economic benefits, including improved animal and human food security, and helping to offset the costs of biofuel production (Stokes, 2015).

Euglena *(Euglena gracilis), one of the microalgae*, has long been known to produce various vitamins and amino acids essential for human health (O'Neill et al., 2015). From our previous in vitro study, the addition of euglena reduced ruminal protozoa population and CH_4_ emission (Aemiro et al., 2016). Based on the results of this in vitro study, the addition of euglena up to 10% of the diet on DM basis has shown considerable improvement in dry matter (DM) and organic matter (OM) digestibility in addition to mitigating CH_4_ emission. The use of fat containing supplements in the diet of animal inhibits methanogenesis by reducing the number of ruminal methanogens (Lillis et al., 2011) and protozoa (Beauchemin, McGinn, Benchaar, & Holtshausen, 2009). However, there is no information available investigating the effect euglena on in vivo CH_4_ emission to support our earlier in vitro study. Thus, we hypothesized that substituting soybean meal with euglena may reduce CH_4_ emission without affecting the performance of animals’. Therefore, this study was conducted to evaluate the effect of substituting soybean meal with euglena in the diet of Corriedale wether sheep on CH_4_ emission and nitrogen efficiency.

## 2 MATERIALS AND METHODS

### 2.1 Animals, experimental design, and dietary treatments

Animal management and sampling procedures of this study were approved by the Obihiro University of Agriculture and Veterinary Medicine Animal Care and Use Committee. Four Corriedale wether sheep with an average body weight of 53.8 ± 4.6 kg and 2 years old were used in a 4 × 4 Latin square design. The wethers were kept in an individual metabolic cage equipped with ventilated respiratory collection hood and fed a basal diet of Guinea grass hay and concentrate mixture twice daily (08:30 and 16:30 hours) based on nutrient requirements of National Research Council (2007). The roughage to concentrate ratio was 70:30. All sheep had free access to clean drinking water and a mineral block. The mineral block consisted of Iron oxide, 1732 mg; Ferric oxide, 1104 mg; Copper sulphate, 377 mg; Cobalt sulphate, 66 mg; Zinc sulphate, 1235 mg; Manganese carbonate, 1046 mg; Calcium iodate, 77 mg; Sodium selenite, 33 mg; and Sodium chloride, 962 g/kg mineral block. The Feed ingredients used in this study were Guinea grass (*Panicum maximum*) hay, soybean meal, and euglena (*Euglena gracilis*). Euglena*,* powder with 100% purity was obtained from Euglena Co. Ltd., Japan. The chemical compositions of euglena, soybean meal, Guinea grass hay, and the dietary treatments are indicated in Table 1.

**Table 1:** Chemical composition of experimental feed ingredients and dietary treatments

	Ingredients (% DM)			Euglena substituting soybean (% DM)				*SEM*	*p* value		
	Hay	Soybean	Euglena	0	33	50	67		L	Q	C
DM (%)	93.58	93.28	93.70	93.57	93.55	93.53	93.49	0.25	0.31	0.87	0.95
OM (%)	91.23	91.23	94.24	91.23	91.54	91.69	91.84	0.13	0.19	0.83	0.93
Ash (%)	8.71	7.74	5.77	8.42^a^	8.22^ab^	8.12^ab^	8.02^b^	0.24	<0.01	0.41	0.73
CP (%)	10.20	43.50	30.90	20.19^a^	18.94^ab^	18.30^b^	17.67^b^	1.05	<0.01	0.21	0.58
EE (%)	3.01	2.08	15.35	2.72^d^	4.07^c^	4.76^b^	5.44^a^	0.72	0.01	0.01	0.07
GE (MJ/kg)	16.83	18.41	21.17	17.30	17.59	17.72	17.86	0.09	0.052	0.70	0.86
NDF (%)	65.45	13.93	0.10	49.99	48.62	479.2	47.23	0.33	0.054	0.68	0.87
ADF (%)	30.18	9.24	0.00	23.90	22.98	22.51	22.05	0.28	0.08	0.75	0.90

Notes

All the data are the average result of chemical analysis conducted in duplicate.

Means within the same row without a common letter differ significantly (*p *<* *0.05).

DM: dry matter; OM: organic matter; CP: crude protein; EE: ether extract; GE: gross energy; NDF: neutral detergent fiber; ADF: acid detergent fiber; *SEM*: standard error of the mean; L: linear; Q: quadratic; C: cubic.

The dietary treatments were: treatment 1, control (70% Guinea grass hay and 30% soybean meal); treatments 2, (70% Guinea grass hay, 20% soybean meal, and 10% euglena); treatment 3 (70% Guinea grass hay, 15% soybean meal, and 15% euglena) and treatment 4 (70% Guinea grass hay, 10% soybean meal, and 20% euglena) per kg DM of the total ration. Euglena powder was thoroughly mixed with soybean meal in each treatment to avoid preference. The result of the chemical composition shows that the crude protein (CP) concentration of the ration decreases linearly (*p *<* *0.01), while gross energy (GE) concentration has shown a tendency to increase linearly (*p *=* *0.052) with increasing substitution of soybean meal with euglena. On the other hand, the ether extract (EE) content of the ration increased linear (*p *<* *0.01), quadratic (*p *<* *0.05), and tended to increase cubic (*p *=* *0.07).

The fatty acid profile of euglena and soybean meal (Table 2), indicated that the proportion of saturated fatty acid (SFA) is higher in euglena (55.2%) compared to soybean meal (20.7%). On the other hand, the proportion of unsaturated fatty acid (UFA) in euglena (38.6%) is lower than soybean meal (78.1%). The amino acid profile indicated that the proportion of each amino acid per the total CP is higher in soybean meal than euglena except for Methionine and Alanine (Table 3).

**Table 2:** Fatty acid profile of euglena and soybean meal

Type of fatty acids	Euglena	Soybean meal
	% of the total fatty acid	% of the total fatty acid
12:0	2.0	–
13:0	5.9	–
14:0	30.4	0.1
14:1	0.4	–
15:0	3.0	–
16:0	10.9	15.2
16:1	5.3	0.2
17:0	0.8	0.2
17:1	1.3	–
18:0	2.1	4.2
18:1	5.4	15.3
18:2n‐6	2.5	52.6
18:3n‐3	1.1	9.8
20:0	–	0.3
20:1	0.4	0.2
20:2n‐6	2.8	–
20:3n‐6	4.9	–
20:3n‐3	0.4	–
20:4n‐6	6.1	–
20:4n‐3	1.3	–
20:5n‐3	1.1	–
22:0	–	0.4
22:4n‐6	3.5	–
22:5n‐6	1.5	–
22:5n‐3	0.4	–
22:6n‐3	0.2	–
24:0	0.1	0.3
SFA	55.2	20.7
UFA	38.6	78.1

SFA: saturated fatty acid; UFA: unsaturated fatty acids.

**Table 3:** Amino acid profile of euglena and soybean meal

Type of Amino acid	Euglena		Soybean	
	% per 100 g euglena on DM basis	% of total CP in euglena	% per 100 g soybean on DM basis	% of total CP in soybean
Arg	2.13	7.27	3.29	7.08
Lys	2.10	7.16	2.93	6.31
Gus	0.79	2.70	1.31	2.82
Phe	1.36	4.64	2.41	5.19
Tyr	1.29	4.40	1.58	3.40
Leu	2.46	8.39	3.65	7.86
Ile	1.18	4.03	2.18	4.69
Met	0.65	2.22	0.65	1.40
Val	1.92	6.55	2.33	5.02
Ala	2.35	8.02	2.06	4.43
Gly	1.50	5.12	2.03	4.37
Pro	1.88	6.41	2.42	5.21
Glu	3.44	11.74	8.57	18.45
Ser	1.24	4.23	2.36	5.08
Thr	1.47	5.02	1.89	4.07
Asp	2.50	8.53	5.45	11.73
Trp	0.53	1.81	0.65	1.40
Cys	0.52	1.77	0.69	1.49

Arg: Arginine; Lys: Lysine; His: Histidine; Phe: Phenylalanine; Tyr: Tyrosine; Leu: Leucine; Ile: Isoleucine; Met: Methionie; Val: Valine; Ala: Alanin; Gly: Glycine; Pro: Proline; Glu: Glutamic acid; Ser: Serine; Thr: Threonine; Asp: Aspartic acid; Trp: Triptophan; Cys: Cysteine.

## 3 EXPERIMENTAL PROCEDURE

The experiment was conducted for 84 days with each 21‐days period consisting of 14‐days of acclimatization followed by a 5‐days digestion trial and two 24‐hr runs in open‐circuit respiration chamber to measure gas exchange. Samples of the offered feed, refusal, feces, and urine were collected and analyzed for nutrient content following standard procedures. Methane emission was quantified by an open‐circuit respiratory system using a hood over the heads of the wethers as described by Takahashi, Chaudhry, Beneke, and Young (1999). Data were collected and entered into a computer through an interface with the analyzers at 1‐min intervals and then automatically standardized at 0°C, 1013 hPa, and zero water vapor pressure.

### 3.1 Feces and urine sample collection and preparation

Feces and urine were collected for 5‐days in each period, and the fecal samples from each treatment were thawed, bulked, mixed, and subsampled. Subsamples were dried at 60°C for 48 hr in a forced‐air oven and ground to pass through a 1‐mm sieve for subsequent laboratory analysis. Urine was collected into buckets containing 100 ml of 100 ml/L (v/v) sulfuric acid to reduce the pH below 3.0 and to prevent bacterial degradation of nitrogen compounds. Approximately 50 ml/L of the urine sample was subsampled and stored at −20°C until needed for analysis.

### 3.2 Analysis of chemical composition

Experimental samples were analyzed for DM by drying at 135°C for 2 hr (930.15), and OM, total ash (942.05) and ether extract (EE) (920.39) were determined according to the procedures of the Association of Official Analytical Chemists (AOAC, 1995). Nitrogen was determined by the Kjeldahl method (984.13) (AOAC, 1995) using an electrical heating digester (FOSS Tecator^TM^ Digester, Tokyo, Japan) and an automatic distillation apparatus (FOSS Kjeltec^TM^ 2100, Tokyo, Japan), and crude protein (CP) was then calculated as the amount of *N* × 6.25. The concentrations of neutral detergent fiber (NDF) and acid detergent fiber (ADF) were measured according to the method described by Van Soest, Roberts, and Lewis (1991) and the results were expressed with residual ash. The gross energy (GE) concentration of the samples was analyzed in a Shimadzu auto‐calculating bomb calorimeter (CA‐4AJ; Shimadzu Corporation, Kyoto, Japan).

### 3.3 Amino acid and fatty acid analysis

Amino acid and fatty acid profile of Euglena and soybean meal samples were analyzed by Japan Food Research Laboratories, Japan. The amino acid composition except for tryptophan was carried out by an automated amino acid analyzer (JLC‐500/V, JEOL Ltd., Tokyo, Japan; Column, LCR‐6 with 4 ×120 mm ID, JEOL, Co. Ltd.). Tryptophan was analyzed by high‐performance liquid chromatography (HPLC, LC‐20AD, Shimadzu, Co. Ltd., Kyoto, Japan; Column, CAPCELL PAK C18 AQ, 4.6 mm ID × 250 mm, Shiseido Co. Ltd., Tokyo, Japan; a detector, Flourospectro photometer, RF‐20Axs, Shimadzu, Co. Ltd., Kyoto, Japan). The mobile phase consisted of perchloric acid and methanol (80:20). The flow rate was 0.7 ml/min and the fluorescence excitation was at 285 nm and 40°C. Fatty acid composition of Euglena was determined by gas chromatography, GC‐1700, Shimadzu Co. Ltd., Japan equipped with FID. The fatty acids were separated on 30 m × 0.25 mm ID, DB‐23 capillary column. Helium was used as a carrier gas at a flow rate of 1.5 ml/min with splitless injection at 250°C and the detector temperature was 250°C.

### 3.4 Calculation

The total CH_4_ gas volume obtained from the open‐circuit respiratory system was converted to its GE value using a conversion factor of 39.54 kJ/L (Brouwer, 1965). Digestible energy (DE) was calculated as the difference between energy intake and fecal energy; energy lost as CH_4_ was the CH_4_ emitted in L/day × 39.54 kJ/L (Brouwer, 1965). Nitrogen retention (g/day) was calculated as the difference between total nitrogen intake in diets and the total fecal and urinary nitrogen, Nitrogen efficiency was calculated as (nitrogen retained divided by the total nitrogen intake) ×100. Total feed efficiency was calculated as (average daily weight gain divided by daily DM intake) ×100.

### 3.5 Statistical analysis

Data obtained from the in vivo study were subjected to ANOVA in a 4 × 4 Latin square design using REG procedure (SAS 2010) with the model: *Yij *= *μ *+ *Ti *+ *eij*, where, *Yij* is the dependent variable; *μ* is the overall mean; *Ti* is the fixed treatment effect; *Ai* is the random animal effect; *Pi* is the random period effect and *eij* is the residual. Dietary treatments were considered fixed whereas sheep and periods were random variables. Total number of observations for intake and digestibility were 320 (treatment [4] × animal [4] × period [4] × data collection days [5]) and for methane emission 128 (treatment [4] × animal [4] × period [4] × data collection days [2]). The standard error of the means was determined using the least squares means procedure (lsmeans option) in SAS (2010). Normality of data was verified and outliers were tested using the REG procedure (SAS Institute, Cary, NC, USA).

## 4 RESULTS

### 4.1 Effect of substituting soybean meal with euglena on intake and digestibility

Nutrient intake at different proportions of soybean meal and euglena in the diets of sheep is indicated in Table 4. Dry matter and OM intake were not influenced by substitution of soybean meal with euglena (*p *>* *0.05), while there was a linear decrease (*p *<* *0.01) in NDF, ADF, and CP intake. On the other hand, GE (MJ/day) intake increased linearly (*p *<* *0.01) with increasing concentration of euglena (Table 4).

**Table 4:** Nutrient intake as a function of the substitution levels of soybean meal with euglena in the diets of Corriedale wether sheep

Variables	Euglena substituting soybean (% DM)				*SEM*	*p* value		
	0	33	50	67		L	Q	C
DM (g/day)	968.57	976.39	973.81	975.1	4.38	0.52	0.58	0.59
OM (g/day)	886.53	895.64	894.25	896.41	2.39	0.24	0.52	0.56
NDF (g/day)	484.22^a^	474.62^ab^	463.64^bc^	460.52^c^	1.27	<0.01	0.55	0.99
ADF (g/day)	231.45^a^	224.30^b^	219.21^c^	215.00^c^	0.60	<0.01	0.28	0.85
CP (g/day)	192.32^a^	181.50^b^	175.80^c^	171.28^d^	0.29	<0.01	<0.01	0.14
GE (MJ/day)	16.75^b^	17.16^a^	17.23^a^	17.39^a^	0.04	<0.01	0.19	0.34

The data are the means of 20 observations (5 days and 4 periods).

Means within the same row without a common letter differ significantly (*p *<* *0.05).

DM: dry matter; OM: organic matter; NDF: neutral detergent fiber; ADF: acid detergent fiber; CP: crude protein; GE: gross energy; *SEM*: standard error of the mean; L: linear; Q: quadratic; C: cubic.

Apparent nutrient digestibility of dietary treatments when euglena substitute's soybean meal at different proportion in the diet of sheep is shown in Table 5. Substitution did not affect DM and GE digestibility (*p *>* *0.05) but it has shown a tendency to reduce linearly (*p* = 0.06 and *p *=* *0.051) for DM and GE, respectively. Organic matter (linear, *p *<* *0.01), NDF (linear, *p *<* *0.05) and ADF (linear, *p *<* *0.01; quadratic, *p *<* *0.05) digestibility reduced significantly with increasing substitution of soybean meal with euglena. The CP digestibility was significantly reduced (linear, *p *<* *0.01; quadratic, *p *<* *0.05) with decreasing concentration of soybean meal.

**Table 5:** Mean values of apparent nutrient digestibility when soybean meal is substituted with euglena at different rates in the Corriedale wether sheep diets (%)

Variables	Euglena substituting soybean (% DM)				*SEM*	*p* value		
	0	33	50	67		L	Q	C
DM (%)	75	74	73	73	0.01	0.06	0.37	0.27
OM (%)	80^a^	79^ab^	77^b^	77^b^	0.20	<0.01	0.31	0.23
NDF (%)	74^a^	73^ab^	71^b^	71^b^	0.30	0.02	0.13	0.16
ADF (%)	69^a^	67^ab^	64^b^	66^b^	0.01	<0.01	0.02	0.28
CP (%)	83^a^	81^b^	78^c^	78^c^	0.30	<0.01	0.03	0.45
GE (%)	75	74	72	73	0.32	0.051	0.35	0.29

The data are the means of 20 observations (5 days and 4 periods).

Means within the same row without a common letter differ significantly (*p *<* *0.05).

DM: dry matter; OM: organic matter; NDF: neutral detergent fiber; ADF: acid detergent fiber; CP: crude protein; GE: gross energy; *SEM*: standard error of the mean; L: linear; Q: quadratic; C: cubic.

### 4.2 Effect of euglena supplementation on CH_4_ emission and energy loss

Table 6 shows the effect of substituting soybean with euglena at a different concentration on CH_4_ emission and energy loss via CH_4_. Methane emission expressed in L/day, g/day, L/kg DM intake, g/kg DM intake and energy loss as CH_4_ (% of GE intake) was reduced linearly and quadratically (*p *<* *0.01) with increasing concentration of euglena in the diet. Fecal energy loss as a proportion of GE intake was in the range of 25%–28% and it was not affected by the concentration of euglena. However, it has shown a tendency to increase linearly (*p *=* *0.051). As a result, the DE remained unaffected by the variation in the proportion of euglena and soybean meal in the diet (Table 7). On the other hand, energy loss via CH_4_ as a proportion of GE intake reduced linearly (*p *<* *0.01), quadratically (*p *<* *0.01), and cubically (*p *<* *0.05).

**Table 6:** Effect of euglena substituting soybean meal in the diet of sheep on Methane emission

Parameters	Euglena substituting soybean (% DM)				*SEM*	*p* value		
	0	330	500	670		L	Q	C
CH_4_ (L/day)	46.38^a^	46.40^a^	40.01^b^	30.52^c^	0.737	<0.01	<0.01	0.62
CH_4_ (g/day)	33.20^a^	33.22^a^	28.65^b^	21.86^c^	0.527	<0.01	<0.01	0.62
CH_4_ E (MJ/day)	1.84^a^	1.84^a^	1.58^b^	1.21^c^	0.029	<0.01	<0.01	0.63
CH_4_ (L/kg DMI)	44.74^a^	44.49^a^	38.23^b^	28.26^c^	0.768	<0.01	<0.01	0.73
CH_4_ (g/kg DMI)	32.03^a^	31.86^a^	27.37^b^	20.30^c^	0.55	<0.01	<0.01	0.73
CH_4_ (ml/min/MBW)	1.57^a^	1.56^a^	1.35^b^	1.03^c^	0.025	<0.01	<0.01	0.65

The data are means of 8 observations (2 days and 4 periods).

Means within the same row without a common letter differ significantly (*p *<* *0.05).

CH_4_: methane; L: liter; g: gram; E: energy; MJ: mega joule; kg; kilogram; DMI: dry matter intake; ml: milliliter; min: minute; MBW: metabolic body weight; *SEM*: standard error of the mean; L: linear; Q: quadratic; C: cubic.

**Table 7:** Effect of soybean meal substitution with euglena on energy loss and saving

Parameters	Euglena substituting soybean (% DM)				*SEM*	*p* value		
	0	33	50	67		L	Q	C
DE intake, MJ/day	12.51	12.72	12.49	12.72	0.07	0.57	0.94	0.23
Energy loss via methane								
% GE intake	11.00^a^	11.00^a^	9.00^b^	7.00^c^	0.06	<0.01	<0.01	0.02
% DE	14.80^a^	14.50^a^	12.70^b^	9.50^c^	0.10	<0.01	<0.01	0.85
Fecal energy								
MJ/day	4.23^b^	4.45^ab^	4.74^a^	4.66^a^	0.05	0.03	0.21	0.38
As a proportion of GE intake	0.25	0.26	0.28	0.27	0. 30	0.051	0.35	0.29

The data are means of 20 observations (5 days and 4 periods).

Means within the same row without a common letter differ significantly (*p *<* *0.05).

DE: digestible energy; MJ: mega joule; GE: gross energy; *SEM*: standard error of the mean; L: linear; Q: quadratic; C: cubic.

### 4.3 The effect of soybean meal substitution with euglena on efficiency of protein utilization

The effect of euglena substitution on protein balance is indicated in Table 8. Crude protein intake was higher for the soybean meal supplemented group (control) and it reduced significantly (linear and quadratic, *p *<* *0.01) with increasing concentration of euglena. Fecal CP loss, as a proportion of CP intake, increased linearly (*p *<* *0.01) and quadratically (*p *<* *0.05) with increasing substitution of soybean meal with euglena. Similarly fecal CP loss (g/day) increased linearly (*p *<* *0.01) and tended to reduce quadratically (*p *<* *0.06). On the other hand, urinary CP loss (g/day and as a proportion of total CP intake) reduced linearly and quadratically (*p *<* *0.01) with increasing concentration of euglena in the sheep diets. The overall total CP loss as a proportion of CP intake reduced significantly (linear, *p *=* *0.01) with increasing euglena concentration. Crude protein efficiency (CP retained/CP intake) increased linearly (*p *<* *0.01). Average daily weight gain and overall feed efficiency remained unaffected (*p *>* *0.05).

**Table 8:** Protein retention, efficiency of utilization, and loss at different proportion of soybean meal and euglena in the diets of Corriedale wether sheep

Variables	Euglena substituting soybean (% DM)				*SEM*	*p* value		
	0	33	50	67		L	Q	C
Fecal CP (g/day)	32.47^b^	35.28^ab^	38.13^a^	37.12^a^	0.46	<0.01	0.06	0.39
Fecal CP (% CP intake)	16.90^c^	19.40^b^	21.80^a^	21.70^a^	0.30	<0.01	0.03	0.37
Urinary CP (g/day)	107.35^a^	93.10^b^	85.62^cb^	77.79^c^	1.25	<0.01	0.25	0.57
Urinary CP (% CP intake)	55.90^a^	51.30^ab^	48.70^bc^	45.30^c^	0. 70	<0.01	0.68	0.69
CP loss (g/day)	139.82^a^	128.38^b^	123.74^bc^	114.90^c^	1.22	<0.01	0.64	0.37
Urinary CP (% total CP loss)	76.70^a^	72.20^b^	68.90^bc^	67.70^c^	0.50	<0.01	0.11	0.82
Fecal CP (% total CP loss)	23.40^c^	27.80^b^	31.20^ab^	32.40^a^	0.50	<0.01	0.11	0.82
CP loss (% CP intake)	72.80^a^	70.80^ab^	70.40^ab^	67.00^b^	0.70	0.01	0.64	0.48
CP retained (g/day)	52.50	53.13	52.05	56.37	1.20	0.38	0.50	0.56
% CP efficiency	27.30^b^	29.30^ab^	29.60^ab^	33.00^a^	0.70	0.01	0.64	0.48
ADG (g/day)	68.75	68.75	70.00	75.00	3.14	0.54	0.74	0.94
Feed efficiency (ADG/kg DMI)	7.13	7.07	7.22	7.70	0.34	0.60	0.74	0.98

The data are means of 20 observations (5 days and 4 periods).

Means within the same row without a common letter differ significantly (*p *<* *0.05).

CP: crude protein; g: gram; CP efficiency: (CP retained divided by CP intake) ×100; ADG: average daily gain; *SEM*: standard error of the mean; L: linear; Q: quadratic; C: cubic.

## 5 DISCUSSION

In the present study, substitution of soybean meal with euglena at different proportion on a basal diet of Guinea grass hay was evaluated on CH_4_ emission, intake and digestibility, energy loss, and nitrogen efficiency of Corriedale wether sheep. The substitution has changed nutrient intake and digestibility, but the overall nitrogen balance and average daily body weight gain remained unaffected. Substitution of soybean meal with euglena has also shown substantial reduction in CH_4_ emission and energy loss through CH_4_. In our previous study, we evaluated the potential of euglena substituting conventional concentrate mixture and the findings indicated that there was improvement in apparent DM and CP digestibility and nutrient efficiency (Aemiro et al., 2017). However, the concentrate mixture used for comparison purpose was lower in digestibility, nitrogen content, and amino acid profile compared with euglena. Because of that reason, a supplemental feed comparable to euglena in terms of quality was considered. Thus, the comparison was made with soybean meal, which is one of the typical protein sources for ruminants with high nitrogen content, high digestibility, and good amino acid profile comparable to euglena. We considered a simple dietary condition in which soybean meal was used as a sole concentrate supplement and substitution was made with euglena to simplify the occurrence of any possible confounding effects due to the presence of multiple ingredients in the concentrate mixture. The potential of commercial application of microalgae are enormous for animal feeds, but despite developments over the years, the amount of commercially available products are still limited (Milledge, 2012).

### 5.1 The effect of euglena supplementation on CH_4_ emission

The amount of carbohydrates fermented in the rumen and the ratio of propionate to acetate produced are the two causes of variation for methane production (Johnson & Johnson, 1995). These authors indicated that dietary lipids affect enteric CH_4_ emission depending on the level of lipid supplementation, the chain length of fatty acids, and the interactions between lipids and the basal diet composition. In the present study, enteric CH_4_ emission expressed as g CH_4_/kg DM intake or L CH_4_/kg DM intake was reduced by up to 37% for animals fed euglena substituting up to 67% of soybean meal in the diet. Similarly, enteric CH_4_ emission expressed as (L/day, g/day or ml/min/metabolic body weight (MBW)) was reduced by up to 34% of the methane emission compared to the control group. This is due to higher concentration of fat in euglena that may reduce ruminal fermentation and consequently affected organic matter and fiber digestibility with a concomitant decrease in CH_4_ production. In support of our findings, a study by Hristov et al. (2013) indicated that the amount of hydrogen produced during the fermentation process was reduced due to fat supplementation resulting in decreased CH_4_ production. Most of the previous studies agree that enteric CH_4_ emissions can be reduced by including fat in the diet of ruminants (Grainger & Beauchemin, 2011; Moate et al., 2011; Patra, 2014; Soliva, Meile, Hindrichsen, Kreuzer, & Machmüller, 2004).

In the current study, increasing the levels of substitution of soybean meal with euglena increased the EE content from 2.7% to 5.4%. With this scenario, the proportion of saturated fatty acid in the diet increased. Among the medium chain saturated fatty acids, myristic acid (C14:0) was the dominant fatty acid that constitutes 30% of the total fatty acid in euglena. The presence of higher proportion of this saturated fatty acid in the diet was responsible for reduction in enteric CH_4_ emission by reducing protozoa activity as evident in our previous in vitro study (Aemiro et al., 2016). In the present in vivo study, enteric CH_4_ emission reduced relatively at a higher proportion per unit of dietary fat inclusion compared to previous reports by Patra (2014). This variation might be associated to the difference in the proportion of forage to concentrate ratio and/or the source of the lipid. In this study, it was observed that energy loss as methane was 11% of the gross energy intake for soybean meal supplemented group and this loss reduced to 7% when euglena substituted soybean meal at the rate of 67% of the supplemental feed. This demonstrates that substituting soybean meal with euglena showed beneficial effect in reducing energy loss via CH_4_ and increased energy efficiency in addition to reducing enteric methane emission. This result is within the expected range reported by Martin, Dubroeucq, Micol, Agabriel, and Doreau *(* 2007), who indicated that CH_4_ loss remains relatively constant (6%–7% of the GE intake) in the diet containing 30%–40% concentrate.

### 5.2 Effect of euglena substitution on intake and digestibility

The influence of dietary fat on feed intake, digestibility, and rumen fermentation vary among studies, which could be associated with type and concentrations of fat, dietary composition, and species of animals (Grainger & Beauchemin, 2011; Machmüller, 2006; Patra, 2014). In the present study, variation in dietary lipid content due to substitution of soybean meal with euglena did not affect intake of DM, OM, CP, GE, or fiber component. This fact was proven in a meta‐analysis study conducted by Patra, 2014; who indicated that addition of fat in diets did not affect DM intake. In addition, previous study by Palmquist and Jenkins (1980) showed that lipid supplementation to the diet may reduce DM intake when the EE content is higher than 7% of the diet on DM basis. From our current study, we observed that apparent digestibility of OM, NDF, and ADF reduced while apparent DM and GE digestibility had shown tendency to reduce. This is in agreement with the findings by Huws et al., 2010; Patra, 2014; who stated that dietary fat affect NDF digestibility due to decreases in protozoa and bacteria population, and activities of fiber degrading enzymes. Similarly the findings of Salem, Krzeminski, Ferlay, and Doreau (1993) also confirmed that dietary lipid supplementation for ruminants often implies a decrease in organic matter digestibility, mainly due to a decrease in fiber digestion in the rumen.

The present study demonstrated that digestibility can be negatively affected by lipid content even at lower than 5.5% of the diet. On the other hand, most of the previous studies indicate that supplementation of lipid reduce apparent digestibility when the level of inclusion is above 7% of the ration. This may be associated to the type and source of the lipids in the diet and forage to concentrate ratio. The observation in this study indicates that with increasing levels of substitution of euglena for soybean meal, the diet becomes richer in saturated fatty acids, compared to soybean meal supplemented group. This was in accordance with the results found in the literature by Weld and Armentado (2015), who observed that supplementation of fat high in medium chain fatty acid (C12, C14) decreased NDF digestibility.

### 5.3 Effect of soybean meal substitution with euglena on energy loss and saving

Supplementation of ruminant with dietary lipids is widely used to increase the energy content of the diet and to enhance energy utilization for improving animal performance (Hess, Moss, & Hule, 2008). In ruminants, the energy intake lost through CH_4_ production has been estimated to vary from 2% to 12% of gross energy intake (Johnson & Johnson, 1995). In our study, despite an increase in the GE intake with increasing concentration of euglena in the diets of sheep, DE intake remains unchanged. This is due to the fact that the fecal energy loss as a proportion of GE intake was not influenced by the levels of euglena substitution. On the other hand, the energy loss through CH_4_ (as a proportion of GE intake) reduced with increasing replacement of soybean meal with euglena.

### 5.4 Effect of soybean meal substitution with euglena on nitrogen balance

The presence of higher concentration of nitrogen in soybean meal supplemented group (control) did not show a better response in terms of nitrogen balance and average daily weight gain compared to the treatment groups where euglena substituted soybean meal at different concentrations. In this study, the majority of the protein loss was encountered though urine (68%–77% of the total CP loss). The result indicates that digestible protein loss was higher in soybean meal supplemented group and this situation was reversed with increasing concentration of euglena. This suggests that efficiency of digestible nitrogen utilization increased with increasing euglena concentration. This might be associated with the presence of higher fat concentration in euglena that might inactivated microbial activity in the rumen and enhanced availability of diversified types of amino acids in the lower digestive tract for absorption. As a result, there was no change in nitrogen balance even though there was a higher concentration of nitrogen in the control group (100% soybean supplemented group). This shows that an increase in lipid content due to increased proportion of euglena in the diet did not affect average daily weight gain. Related studies indicate that decrease in the number of protozoa is generally associated with a reduction in bacterial nitrogen recycling in the rumen when lipid containing supplements are provided for ruminants (Morias, Berchielli, & Reis, 2006). Thus, an increase in the number of gram negative bacteria and a decrease in ammonia concentration occur (Jouany, 1996), with a possible increase in the flow rate of rumen solids (Czerkawski, Christie, Breckenridge, & Hunter, 1975). These effects contribute toward an increase in the efficiency of protein utilization in the lower part of the digestive tract. Previous findings confirmed that the presence of dietary compounds (including oil) can reduce rumen fermentation and enhance flow of dietary protein escaping ruminal degradation (Tandon, Siddique, & Ambwani, 2008). These authors elaborated that the protein that enter in to the lower digestive tract is absorbed in the form of amino acids following enzymatic digestion. In general, replacing soybean meal with euglena in the diet of sheep had shown better response in the efficiency of nitrogen utilization.

## 6 CONCLUSION

Substituting soybean meal with euglena in the diet of sheep reduced CH_4_ emission, and reduced energy loss as CH_4_ and increased efficiency of digestible nitrogen utilization without any effect on the overall nitrogen balance and average daily weight gain despite a higher nitrogen concentration in the control group.
